# Differential Gene Expression and Allele Frequency Changes Favour Adaptation of a Heterogeneous Yeast Population to Nitrogen-Limited Fermentations

**DOI:** 10.3389/fmicb.2020.01204

**Published:** 2020-06-15

**Authors:** Eduardo I. Kessi-Pérez, Belén Ponce, Jing Li, Jennifer Molinet, Camila Baeza, David Figueroa, Camila Bastías, Marco Gaete, Gianni Liti, Alvaro Díaz-Barrera, Francisco Salinas, Claudio Martínez

**Affiliations:** ^1^Departamento de Ciencia y Tecnología de los Alimentos, Universidad de Santiago de Chile (USACH), Santiago, Chile; ^2^Centro de Estudios en Ciencia y Tecnología de Alimentos (CECTA), Universidad de Santiago de Chile (USACH), Santiago, Chile; ^3^Escuela de Ingeniería Bioquímica, Pontificia Universidad Católica de Valparaíso, Valparaíso, Chile; ^4^Université Côte d’Azur, CNRS, INSERM, IRCAN, Nice, France; ^5^State Key Laboratory of Oncology in South China, Collaborative Innovation Center for Cancer Medicine, Sun Yat-sen University Cancer Center, Guangzhou, China; ^6^Millennium Institute for Integrative Biology (iBio), Santiago, Chile; ^7^Instituto de Bioquímica y Microbiología, Facultad de Ciencias, Universidad Austral de Chile (UACH), Valdivia, Chile

**Keywords:** *Saccharomyces cerevisiae*, heterogeneous yeast population, selection experiments, nitrogen consumption, fermentation process

## Abstract

Alcoholic fermentation is fundamentally an adaptation process, in which the yeast *Saccharomyces cerevisiae* outperforms its competitors and takes over the fermentation process itself. Although wine yeast strains appear to be adapted to the stressful conditions of alcoholic fermentation, nitrogen limitations in grape must cause stuck or slow fermentations, generating significant economic losses for the wine industry. One way to discover the genetic bases that promote yeast adaptation to nitrogen-deficient environments are selection experiments, where a yeast population undergoes selection under conditions of nitrogen restriction for a number of generations, to then identify by sequencing the molecular characteristics that promote this adaptation. In this work, we carried out selection experiments in bioreactors imitating wine fermentation under nitrogen-limited fermentation conditions (SM60), using the heterogeneous SGRP-4X yeast population, to then sequence the transcriptome and the genome of the population at different time points of the selection process. The transcriptomic results showed an overexpression of genes from the NA strain (North American/YPS128), a wild, non-domesticated isolate. In addition, genome sequencing and allele frequency results allowed several QTLs to be mapped for adaptation to nitrogen-limited fermentation. Finally, we validated the *ECM38* allele of NA strain as responsible for higher growth efficiency under nitrogen-limited conditions. Taken together, our results revealed a complex pattern of molecular signatures favouring adaptation of the yeast population to nitrogen-limited fermentations, including differential gene expression, allele frequency changes and loss of the mitochondrial genome. Finally, the results suggest that wild alleles from a non-domesticated isolate (NA) may have a relevant role in the adaptation to the assayed fermentation conditions, with the consequent potential of these alleles for the genetic improvement of wine yeast strains.

## Introduction

The yeast *Saccharomyces cerevisiae* is one of the most intensively studied microorganisms and a biological model with diverse biotechnological applications, being the first eukaryote to be completely sequenced more than 20 years ago (Goffeau et al., 1996). From then, various other sequencing efforts have been made, focused on individual genes (Fay and Benavides, 2005) or complete genomes (Liti et al., 2009; Strope et al., 2015; Yue et al., 2017; Peter J. et al., 2018), generating a comprehensive view of yeast genetic variation. Interestingly, one of the first yeast genome sequencing attempts was executed more than 10 years ago, unveiling its population structure and demonstrating the presence of five lineages: Malaysian (MA), North American (NA), Sake (SA), West African (WA), and Wine European (WE) (Liti et al., 2009).

The identification of lineages in the species allowed the selection of representative strains from each of them: UWOPS03-461.4 (MA), YPS128 (NA), Y12 (SA), DBVPG6044 (WA), and DBVPG6765 (WE) (Liti et al., 2009). Among them, NA and MA strains were isolated from non-domesticated environments (soil and bertam palm nectar, respectively), not being directly associated with human activities such as industrial fermentations (Liti et al., 2009; Peter J. et al., 2018). Despite MA strain, which is reproductively isolated from the other strains (Cubillos et al., 2009), the other four representative strains have become a powerful resource for studying the genetic basis of *S. cerevisiae* natural variation (Cubillos et al., 2011; Liti and Louis, 2012; Villalobos-Cid et al., 2019). These strains have been extensively used as founder (parental) strains for recombinant yeast populations, which have been used in genetic studies addressing the causative alleles of phenotypic variation through various approaches, such as quantitative trait loci (QTL) mapping, selection experiments and RNAseq (Parts et al., 2011; Kessi-Perez et al., 2016; Cubillos et al., 2017; Quispe et al., 2017).

Selection experiments, where a yeast population is grown under selective conditions, are also commonly known as evolve and resequencing experiments (Long et al., 2015). In these experiments, the initial yeast population contains individuals with homogeneous (clonal) or heterogeneous genetic backgrounds, which are under a selective pressure (e.g., temperature, chemicals, starving, etc.) for a certain number of generation and where different time points of the selection process are sequenced, allowing the accurate determination of the molecular signatures favouring adaptation in the population (Burke et al., 2014). Depending of the initial population (clonal o heterogeneous genetic background), adaptation process is mainly driven by *de novo* mutations or the existing genetic variation present in the population, respectively (Burke et al., 2014; Li et al., 2019). For instance, a heterogeneous yeast population has been obtained from the NAxWA cross using successive rounds of sporulation and random mating (intercrossing), generating an F12 population of millions of segregant with highly recombined genomes (Parts et al., 2011). This population was grown under heat selection (40°C), allowing the identification by sequencing of several genomic regions with high allele frequency that favour adaptation to this condition (Parts et al., 2011). Similarly, this F12 population was grown in different chemical stressors, showing that adaptation process involved a combination of various genetic innovations such as *de novo* mutations, copy number variation (CNV), loss of heterozygosity and changes in allele frequency (Vazquez-Garcia et al., 2017).

The genetic diversity of yeast populations utilized in selection experiments has been increased by inclusion of multiple parental strains (Cubillos et al., 2013). In this sense, the four above mentioned representative strains of yeast lineages (NA, SA, WA, and WE) were outcrossed for 12 generations to generate the SGRP-4X population, composed of 10–100 million random segregants (Cubillos et al., 2013). The SGRP-4X population has been used for the identification of genetic variants underlying stress resistance (heat, arsenite, and paraquat) (Cubillos et al., 2013). Undergoing this yeast population to selection experiments under heat stress condition, sequencing different time points across the experiment, revealed changes in the population allele frequency, permitting to map several QTLs related to heat resistance (Cubillos et al., 2013). Recently, the SGRP-4X population has been assayed under chemical selection (rapamycin and hydroxyurea), which allowed the identification of several QTLs and *de novo* variants implicated in the resistance to these chemicals (Li et al., 2019). Therefore, selection experiments using the SGRP-4X population represent an outstanding resource to identify the genetic hallmarks permitting yeast adaptation to multiple environments, opening the chance to apply this experimental approach to fermentations with nitrogen-limited conditions.

On the other hand, *S. cerevisiae* is a species of industrial importance, being the main microorganism responsible for wine production by carrying out the alcoholic fermentation of the grape must (Pretorius, 2000). Various phenotypes related to the fermentation process are quantitative traits and have been studied by QTL mapping, such as: resistance to fungicides and fermentation kinetics (Kessi-Perez et al., 2016); ethanol production, residual sugar, and acidity (Ambroset et al., 2011; Salinas et al., 2012; Steyer et al., 2012; Trindade de Carvalho et al., 2017; Eder et al., 2018); and nitrogen sources consumption and utilization (Brice et al., 2014a; Jara et al., 2014; Cubillos et al., 2017; Brice et al., 2018; Kessi-Perez et al., 2019a; Molinet et al., 2019). Other studies finding the genetic bases of nitrogen consumption include transcriptomics (Mendes-Ferreira et al., 2007; Contreras et al., 2012; Brice et al., 2014b; Barbosa et al., 2015), massive hemizygote analysis (Gutierrez et al., 2013), deletion collections (Peter J. J. et al., 2018), and allele specific expression (ASE) (Cubillos, 2016; Salinas et al., 2016).

In this sense, nitrogen sources consumption is a phenotype of special biotechnological interest because, while carbon sources tend to be found in excess in the grape must, nitrogen sources could be in limiting concentrations (Barbosa et al., 2009). Nitrogen could be present in various forms in the grape must (amino acids, ammonium, urea, polyamines, amines, etc.), but not all of them are assimilated in the same way by *S. cerevisiae*, with ammonium and amino acids as the most consumed nitrogen sources and its amounts collectively known as Yeast Assimilable Nitrogen (YAN) (Vilanova et al., 2007). Moreover, nitrogen sources are classified as “preferred” when sustaining high growth rate (such as glutamine, glutamate, asparagine, and ammonium) and “less preferred” (such as arginine) or “non-preferred” (such as proline, allantoin, and urea) when allows slow growth rate (Zhang et al., 2018).

Importantly, nitrogen sources are key factors regulating the biomass content during the fermentation process, directly impacting the fermentation rate (Varela et al., 2004). Thus, nitrogen deficiencies can lead to stuck or sluggish fermentations, reducing the fermentation rate and generating economic losses for the industry (Taillandier et al., 2007). One common oenological practice to cover the low nitrogen contents is the addition of ammonium salts to the grape must, leaving large quantities of residual nitrogen in the wine, which in turn may result in the production of undesired compounds such as hydrogen sulphide and ethyl carbamate (Mendes-Ferreira et al., 2004; Garde-Cerdán and Ancín-Azpilicueta, 2008). Therefore, understanding the genetic basis of yeast adaption to nitrogen-limited fermentation conditions is an important goal to generate biotechnological solutions for stuck fermentations caused by nitrogen deficiencies in the grape must.

In the present study, we performed nitrogen-limited cultures in bioreactor mimicking wine fermentation conditions, utilizing the SGRP-4X population. During the selection experiments, we sequenced the transcriptome (RNAseq) and genome (DNAseq) of the yeast population. The RNAseq analysis revealed a set of overexpressed genes in the yeast population under nitrogen-limited conditions, where most of the overexpressed alleles came from the NA genetic background. Furthermore, the genome sequencing of the yeast population allowed the identification of several QTLs related to the adaptation to nitrogen-limited fermentations. From these QTLs, we validated *ECM38* allele from the NA strain as responsible for yeast higher growth efficiency under nitrogen-limited conditions. Overall, the results showed a complex pattern of molecular signatures affecting the adaptation of the yeast population to nitrogen-limited fermentations, including differential gene expression, allele frequency changes and loss of the mitochondrial genome. Finally, the results suggest that alleles from the wild NA strain may have an important role in yeast adaptation to the assayed fermentation conditions, opening the possibility to exploit this genetic diversity for biotechnological applications in wine fermentation.

## Materials and Methods

### Yeast Strains and Media

Parental strains used in this work correspond to stable haploid versions of strains representative of four lineages previously described for *S. cerevisiae* (Liti et al., 2009). These strains are YPS128 (North American, “NA”), Y12 (Sake, “SA”), DBVPG6044 (West African, “WA”), and DBVPG6765 (Wine/European, “WE”) (Cubillos et al., 2009). SGRP-4X population correspond to an intercrossed recombinant population composed by 10–100 million segregants derived from these four parental strains (Cubillos et al., 2013).

Biomass generation in Petri dishes were performed using YPDA (1% yeast extract, 2% peptone, 2% dextrose/glucose, and 2% agar) medium. Experiments in bioreactors were performed using synthetic musts (SM), which mimic natural grape musts but with defined compositions and adjusted pH to 3.3, prepared as previously described (Kessi-Perez et al., 2016). The composition of nitrogen source in the SM is 40% ammonium and 60% amino acids, and its concentration was modified at two different levels: 60 mg/L (SM60) and 300 mg/L (SM300) of YAN, which correspond to limiting and non-limiting nitrogen conditions, respectively (Kessi-Perez et al., 2019a; Molinet et al., 2019).

### Bioreactor Experiments in Batch and Continuous Cultures for Parental Strains

In bioreactor batch experiments, a pre-inoculum was made in 10 mL of culture medium (SM300) and incubated at 28°C for 48 h without agitation. The inoculum for bioreactor was prepared in 500 mL Erlenmeyer flasks with 100 mL of culture medium (SM300 or SM60) and 1 mL of the respective pre-inoculum, which was incubated at 28°C and 100 rpm in an orbital shaker incubator (Labtech model LSI-3016R) for 24 h, until a concentration of 1 g/L was reached [determined by optical density at 600 nm (OD_600_)]. Then, a 10% v/v of the inoculum was transferred to a 2 L Biostat B bioreactor (Sartorius, Germany), using a working volume of 1 L, which was operated at 28°C with a constant agitation rate of 100 rpm at pH 3.3, with pH controlled by the automatic addition of 2N NaOH. Anaerobic conditions were maintained by adding nitrogen pulses at 1.6 mL/min of flow rate in periods of 30 min five times per day.

In continuous culture experiments, the bioreactor was inoculated and operated in batch mode until a concentration of 1.8 g/L was reached, which corresponds to 75% of biomass concentration reached in batch culture. Then, the bioreactor was operated in continuous culture mode with a dilution rate (D) value of 0.06 h^–1^. The working volume was kept constant by withdrawing culture broth through a continuously operated peristaltic pump (Masterflex L/S model). It was considered that the chemostat reached steady state conditions when the biomass concentration remained constant (<10% variation) after at least three residence times (τ). Three sampling points were withdrawn from the bioreactor for analysis, corresponding to different modes of operation: at the beginning of batch culture (*T*_0_), at the beginning of continuous culture (*T*_1_) and after reaching steady state of continuous culture (*T*_2_).

### Bioreactor Experiments in Batch and Continuous Cultures for the SGRP-4X Population

In bioreactor batch experiments, two YPDA Petri dishes were plated with 100 μL of a −80°C-stored cryotube containing the SGRP-4X population and incubated at 25°C for 48 h. The inoculum for bioreactor was prepared in 500 mL Erlenmeyer flasks with 100 mL of culture medium (SM300 or SM60) and 150 mg of biomass from the Petri dishes. Then, the bioreactor was inoculated and operated as above-mentioned for the batch cultures of the parental strains.

In continuous culture experiments, the bioreactor was inoculated and operated in batch mode until a concentration of 1.8 g/L was reached. Then, the bioreactor was operated in continuous culture mode with a D value of 0.10 h^–1^ in the same way abovementioned for parental strains, also obtaining the three sampling points at the beginning of batch culture (*T*_0_), at the beginning of continuous culture (*T*_1_) and after reaching steady state of continuous culture (*T*_2_). Samples were rapidly frozen using liquid nitrogen and then stored in glycerol at −80°C prior DNA or RNA extraction. The bioreactor experiments with the SGRP-4X population were carried out in three independent biological replicas for each medium (SM300 and SM60).

### Microculture Fermentations

In the *T*_1_ and *T*_2_ sampling points of the selection experiments, individual colonies were isolated by dilution and plating for phenotypic analysis using growth curves (Vazquez-Garcia et al., 2017; Li et al., 2019). The isolated colonies were phenotyped under fermentative conditions (SM300 and SM60) in microculture conditions by monitoring the OD_600_ of the cells using 30 min intervals in a Synergy HTX microplate reader (Biotek, United States). Relative fitness variables (growth parameters) for each strain were calculated as previously described (Kessi-Perez et al., 2019b). Briefly, efficiency of proliferation, rate of proliferation and lag time of proliferation were extracted from high-density growth curves using Gompertz growth equation (Yin et al., 2003). Statistical analysis of these parameters consisted in Welch two sample *t*-tests, which were performed using R software (R-Core-Team, 2013). All microculture experiments were carried out in three independent biological replicas.

### HPLC Analysis

To evaluate the concentration of YAN, proline, ethanol, glucose, and fructose from samples obtained in bioreactor continuous cultures for parental strains fermentations, we utilized a Shimadzu Prominence HPLC equipment (Shimadzu) using a Bio-Rad HPX –87H column according to (Nissen et al., 1997). The concentration of each amino acid was measured as previously described (Gomez-Alonso et al., 2007).

### Nucleic Acid Extraction and Sequencing

Genomic DNA was extracted from *T*_0_, *T*_1_, and *T*_2_ sampling time points of the selection experiments. Cultures were harvested by centrifugation and cells were treated with 10 units of Zymolyase (Seikagaku Corporation, Japan) for 30 min at 37°C. Then, genomic DNA was extracted using the MasterPure Yeast DNA Purification Kit (Epicentre-Illumina, United States) according to the supplier’s instructions. Total RNA was extracted from the *T*_2_ time point of selection experiment using the E.Z.N.A. Total RNA Kit I (OMEGA, United States) according to the supplier’s instructions. The RNA solution was treated with DNase I (Promega) to remove genomic DNA traces and recovered using the GeneJET RNA Cleanup and Concentration Micro Kit (Thermo Scientific, United States). Purified DNA and RNA concentrations were determined using an UV-Vis spectrophotometer EPOCH equipment (Biotek, United States) and verified by 1.5% agarose gels. Finally, DNA and RNA sequencing (including library preparation) were performed by Illumina HiSeq 4000 sequencing technology, obtaining paired-end read of 100 bp through the service of the Beijing Genomic Institute (BGI, China).

### RNAseq Data Analysis and Validation

The initial RNAseq data analysis was performed using Geneious Prime 2019.0.3 (Biomatters, New Zealand). The reads obtained from RNAseq were mapped to the reference genome (S288c, release R64-1-1), obtained from the SGD (*Saccharomyces* Genome Database^1^), using the TopHat algorithm (Trapnell et al., 2009). Differential gene expression analyses were performed using the DESeq2 software under default settings (Love et al., 2014), considering a statistical cut-off value of “absolute confidence −log_10_ adjusted *p*-value” ≥2. Enriched functional analysis were performed with the ShinyGO v.061 tool^2^ (Ge et al., 2019) for GO Biological Process and Kyoto Encyclopedia of Genes and Genomes (KEGG) categories, considering a *p*-value cut-off (FDR) of 0.05. Evaluation of the parental origin of the alleles was performed by quantifying the number of reads that aligned only with one of the four reference parental genomes (NA, SA, WA, or WE), using the Geneious Prime 2019.0.3 reads mapper (Biomatters, New Zealand). Parental genomes information was obtained from Yue et al. (2017). Statistical analysis for each distribution consisted in Chi-square tests, which were performed using R software (R-Core-Team, 2013).

Posteriorly, RNAseq reads were once again aligned to the *S. cerevisiae* S288c reference genome (Release R64-1-1) following the manual of STAR (Dobin et al., 2013). Variants were called by FreeBayes v0.9.10-3-g47a713e (Garrison and Marth, 2012) and filtered by quality (>30) and depth (>10). For the differentially expressed genes, we used parent-specific markers (SNPs) to distinguish parent-specific expression alleles of these genes and calculate their frequencies. Any parent-specific expression allele with its frequency higher than 95% quantile or lower than 5% quantile of all the alleles in the 187 genes was considered as significant. The same pipeline was applied to investigate the parent-specific expression alleles for the genes at each QTL.

The expression levels obtained by RNAseq were confirmed using the destabilized version of the firefly luciferase reporter gene (Rienzo et al., 2012). The coding sequences of the selected genes (*BAP3*, *DAL1*, *DAL5*, *DAL80*, *GAP1*, *PUT4*, and *UGA4*) were replaced by luciferase, measuring the endogenous promoter activity of each gene under microculture conditions in SM300 and SM60 (Salinas et al., 2016; Kessi-Perez et al., 2019b). Statistical analysis consisted in independent ANOVA and Tukey’s tests, which were performed using R software (R-Core-Team, 2013).

### DNAseq Data Analysis, Quantitative Trait Loci Mapping, and Validation

The sequencing reads were aligned to the *S. cerevisiae* S288c reference genome (Release R64-1-1). Read mapping was carried out with BWA v0.7.16 (Li and Durbin, 2009). Local realignment was performed by GATK. The variants were called by GATK HaplotypeCaller using the default setup. We counted the read numbers covering each segregating site (Cubillos et al., 2013) and estimated the parental allele frequency accordingly. We performed *de novo* mutation calling for each sequenced sample using two different algorithms: GATK 2.1-5-gf3daab0 (DePristo et al., 2011) and FreeBayes v0.9.10-3-g47a713e (Garrison and Marth, 2012). The variants first needed to pass the default filters of the algorithms. Then, we only used the intersect calls of both algorithms and further subtracted the pre-existing variation of the four parents. It is also required each variant to be on a locus with >10 reads in total and >6 reads reporting the variant allele. Finally, we used Ensembl Variant Effect Predictor (VEP) to annotate the mutations (McLaren et al., 2016). We excluded regions with repetitive sequences such as near chromosome ends, which are prone to false positives due to the rich repetitive sequence content. We used samtools 1.7-12-g17a2483 to calculate sequencing depth at each site and used a 10-kb window (step size = 0) to measure the depth across the genome.

Given our allele frequency estimates, we carry out two different approaches to localize QTLs. In the first one, we used a 10-kb sliding window with a 2-kb step size to localize the QTLs. For each population, we compared the allele frequency changes in a window *i* between each time point *t* and *T*_0_ $\left(\Delta \,x_{i}^{j}\,\left(t\right)=x_{i}^{j}\,\left(t\right)-x_{i}^{j}\,\left(0\right),j=\left\{W\,A,N\,A,W\,E,S\,A\right\}\right)$. If there is selection on pre-existing variants, we expect a steady increase of favoured parental allele frequency in the regions under selection as selection proceeds. Therefore, for each time point, we calculated the *z*-score of allele frequency changes compared with *T*_0_ in each population: $z_{\Delta \,x}=\left(\Delta \,x_{i}^{j}-\mu_{\Delta \,x}\right)/\sigma_{\Delta \,x}$. Here, $\Delta \,x_{j}^{i}$ and σ are the mean and standard deviation of $\Delta \,x_{j}^{i}$ in all the populations evolved in the low nitrogen condition at a certain time point. The *z*-score square reflects the allele frequency deviation from *T*_0_. We searched for regions with *z*-score square higher than 99 or 95% quantile for each time point. If the examined regions pass these cut-offs across all the time points in the low nitrogen condition, but not in the control condition, they are assumed to be QTLs. In addition, the z-score squares are required to increase from *T*_1_ to *T*_2_ for the QTLs.

We then used an alternative approach to map QTLs, in which we performed an ANOVA on square root arcsin transformed allele frequencies by linear model and treated the time point as a factor, according to (Burke et al., 2014). Then, we adjusted the *p*-value for multiple test by using the *p*_*adjust*_ function in R (Broman et al., 2003). The adjusted *p*-value was transformed into −log_10_(*p*) to make the plot for genome-wide QTL mapping. We use the 99% quantile of −log_10_(*p*) as cut-off to localize QTLs and filter out those that were also identified in the control condition.

Finally, for validation of *ECM38*, reciprocal hemizygous strains were generated by deletion of the candidate gene using *URA3* as selectable marker in the parental strains. Then, the strain carrying the deletion was crossed with the parental strain of opposite mating type containing the unmodified allele of the candidate gene (Salinas et al., 2012; Jara et al., 2014; Kessi-Perez et al., 2016). The ploidy of the hemizygous strains was confirmed by *MAT* locus PCR (Huxley et al., 1990).

## Results

### Selection Experiment in the SGRP-4X Population Under Fermentative Conditions

Initially, we selected NA strain (non-domesticated) and WE strain (domesticated), two of the four parental strains of the SGRP-4X population, to perform batch cultures in two different SM, with sufficient (SM300) and limited (SM60) nitrogen content, and then determine the basic operating conditions of the bioreactor. The results showed a higher (although not statistically significant) specific growth rate (*μ*_max_) for the NA strain in SM60 compared to the WE strain, and no differences in SM300 (Table 1 and Supplementary Figure 1). Then, a continuous culture was carried out in SM300 with the two parental strains (NA and WE), using the same operational parameters as in the case of the previous batch culture, applying a *D* value of 0.06 h^–1^. To corroborate that fermentative conditions were achieved, different fermentation metabolites were analysed by HPLC at three different time points of a continuous culture fermentation carrying a mixture of NA and WE strains, with special interest in confirming that proline was not consumed as evidence of anaerobic metabolism (Table 2). Regarding carbon sources, glucose, and fructose were consumed during the culture with the subsequent increase of ethanol from 0 to almost 7% v/v (Table 2). While in a traditional batch fermentation higher levels of ethanol production and lower levels of residual sugars are expected, in a continuous culture ethanol is continuously being removed and sugar being added, which explains the obtained values (including the increase in sugar from *T*_1_ to *T*_2_).

**TABLE 1:** Specific growth rate (*μ*_max_) for NA and WE strains in batch fermentations.

**Medium**	**Strain**	***μ*_max_ (h^–1^)^a^**
SM60	NA	0.057
	WE	0.033
SM300	NA	0.065
	WE	0.057

*^*a*^μ_*max*_ calculated using Gompertz model (Yin et al., 2003).*

**TABLE 2:** Metabolites analysis from the mixture of parental strains under continuous culture fermentation in SM300.

**Time**	**YAN (mg/L)**	**Proline (mg/L)**	**Ethanol (% v/v)**	**Glucose (g/L)**	**Fructose (g/L)**
*T*_0_	270.6	569.3	N.D.	116.6	118.5
*T*_1_	75.8	953.5	2.3	37.9	55.7
*T*_2_	16.5	801.7	6.9	51.5	87.1

*N.D., Not determined (value under the detection limit of the method).*

For its part, in terms of the nitrogen sources, while YAN decreased during the continuous culture, proline increased, since it is not consumed under fermentative conditions (Table 2). This observation is concomitant with previous experiments carried out in our laboratory, where an increase in proline concentration was measured in 12 mL fermentations. Importantly, the initial proline is not consumed, to which intracellular proline is added due to cell death as the fermentation progresses. Altogether, these results allowed us to set up the general operational conditions for batch and continuous culture fermentations.

**FIGURE 1:**
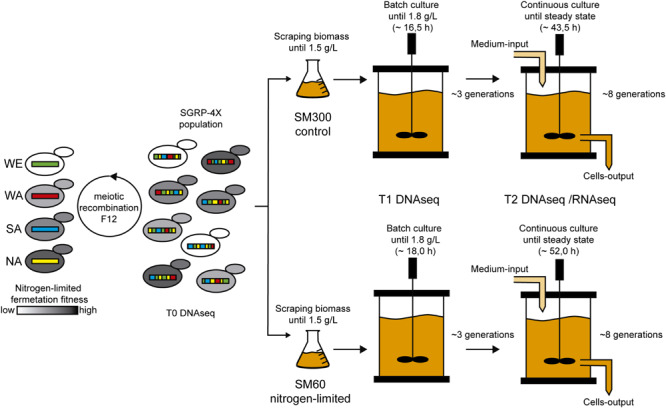
Selection experiment design. The SGRP-4X population (*T*_0_) was subjected to selection in SM60 (low nitrogen) and SM300 (control) culture conditions. For this, the population was scraped from solid plates, grown in cultures flasks until 1.5 g/L and then used for bioreactor inoculation, which operates as batch culture. Once the biomass reached 1.8 g/L, a continuous culture was initiated in the bioreactor keeping constant the nutrients concentration, the population biomass and the selective pressure acting on the population. Two time points of sampling were selected during the selection experiment: just before the beginning of the continuous culture (*T*_1_) and at the end of this regimen (*T*_2_). All the time points (*T*_0_, *T*_1_, and *T*_2_) were subjected to DNA sequencing (DNAseq), and *T*_2_ was also subjected to RNA sequencing (RNAseq).

With all the operational parameters tuned, we performed the selection experiment using the SGRP-4× population composed by 10–100 million segregants with highly recombined genomes (Figure 1). The selection experiment included a batch fermentation step to determine the *μ*_max_ (0.13 h^–1^ in SM300) and for biomass production of the SGRP-4X population (Supplementary Figure 2). The differences in kinetic behaviour respect to the parental strains (Supplementary Figure 1) may have arisen from the different regimes of inoculation for the bioreactor, liquid for parental strains and solid for the SGRP-4X. Once the batch fermentation concentration reached 1.8 g/L, we performed the continuous culture fermentation, using a *D* value of 0.10 h^–1^, which correspond to 3/4 of the *μ*_max_ determined in the batch fermentation. These continuous culture fermentation settings allowed us to maintain constant growth rate of the population. The continuous culture fermentation was stopped after 46 h, reaching a steady state biomass concentration of 1.22 ± 0.05 g/L and 2.77 ± 0.03 g/L in SM60 and SM300, respectively (Supplementary Figure 3). The SM60 continuous culture is limited by nitrogen, thereby it was expected to reach a lower biomass concentration, since the biomass concentration depends directly on the limiting nutrient. During the selection experiment, we took samples at three time points: before selection (*T*_0_), at the beginning of the continuous culture fermentation (*T*_1_), and after reaching steady state of the continuous culture fermentation (*T*_2_) (Figure 1).

Finally, we confirmed that selection process was occurring by isolating 90–96 individual colonies from *T*_1_ to *T*_2_ time points of the three biological replicas of both SM60 and SM300 selection experiments. The isolated colonies were phenotyped by microculture experiments in SM300 and SM60, extracting three growth parameters (efficiency, rate and lag time of proliferation) from the growth curves. The results obtained indicate that selection indeed occurred, since we observed a shift in the mean values and variances of the growth parameters after selection (Supplementary Figure 4). Then, the samples of the selection experiment were analysed by genome (DNAseq) and transcriptome (RNAseq) sequencing (Figure 1).

### Non-domesticated Alleles Are Overexpressed Under Nitrogen-Limiting Conditions

Analysis of RNAseq data corroborated that selection experiment technically sound, as evidenced by the respective PCA plot, volcano plot and heat map (Supplementary Figures 5–7). From the global RNAseq expression analysis (Supplementary Table 1), we considered a cut-off value of “absolute differential expression log_2_ ratio” ≥2 (i.e., the highest differentially expressed genes), obtaining 111 down-regulated and 76 up-regulated genes in SM60 respect to SM300 (Supplementary Figure 6A and Supplementary Table 2). Among the down-regulated genes, many of them are involved in nitrogen uptake and metabolism, especially in the uptake of preferred nitrogen sources (like glutamine and ammonium) and biosynthesis of nitrogen compounds (amino acids, pyrimidines, and pyridoxine). In addition, other cellular processes represented among the down-regulated genes include thiamine metabolism (biosynthesis and uptake) and translation (ribosomal proteins, rRNA processing, etc.), and to a lesser extent iron uptake and homoeostasis (Supplementary Tables 2–5). On the contrary, among the up-regulated genes, many of them are also involved in nitrogen uptake and metabolism, but in this case of less and non-preferred nitrogen sources such as allantoin, arginine, proline, and urea (Supplementary Tables 2–5). Additionally, other cellular processes represented among the up-regulated genes are sporulation, mating, stress response and phosphate metabolism (Supplementary Tables 2–5).

**FIGURE 2:**
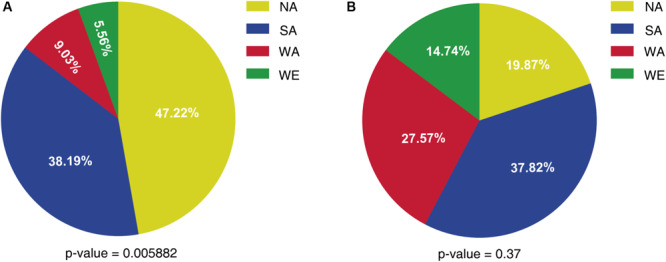
Allele frequency of a subset of differentially expressed genes in SM60. The percentage of alleles from each parental strain is shown for **(A)** SM60 up-regulated genes and **(B)** SM60 down-regulated genes. Statistical analysis for each distribution consisted in Chi-square tests; the obtained *p*-values are shown in each case.

We then considered a stricter cut-off value of “absolute differential expression log_2_ ratio” ≥3, which left 26 down-regulated and 24 up-regulated genes in SM60 respect to SM300 (Supplementary Figures 6B, 7 and Supplementary Table 5). This subset includes various subtelomeric genes according to the reference genome, including more than one gene of *THI*, *SNO*, *SNZ*, and *DAL* families, as well as genes with CNV, all of them located in subtelomeric regions (Supplementary Table 5). Using this set of 50 differentially expressed genes, we evaluated the parental origin of the alleles (NA, SA, WA, or WE) in the SM60 replicas by mapping the reads against the parental genomes. Surprisingly, among the SM60 up-regulated genes the alleles from the non-domesticated (NA) strain showed a higher frequency, while the less frequently alleles were from the domesticated (WE) origin (Figure 2 and Supplementary Table 5). When considering the SM60 down-regulated genes, the distribution of alleles more frequently represented among the four parental origins is not statistically different from the expected even distribution of 25% for each strain (Figure 2 and Supplementary Table 5). In order to confirm these results, we analysed the ASE for the 187 differentially expressed genes in the three replicas of the SM60 selection experiments. However, only a few genes showed a consistent pattern of ASE across the experimental replicas for each genetic background (Supplementary Figure 8).

**FIGURE 3:**
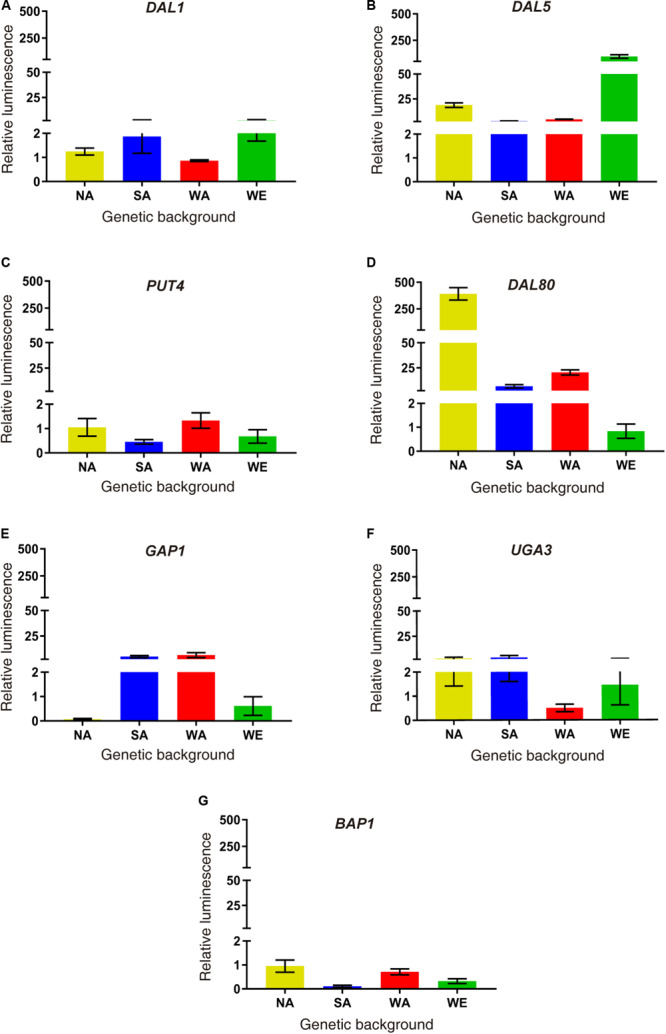
Molecular validations of SM60 up-regulated genes. Luminescence validations were performed for genes **(A)** *DAL1*, **(B)** *DAL5*, **(C)** *PUT4*, **(D)** *DAL80*, **(E)** *GAP1*, **(F)** *UGA3*, and **(G)** *BAP1*. In each case, the area under curve (AUC) was extracted for each luminescence curve (in SM60 and SM300), and then the “relative luminescence” between SM60 and SM300 (SM60/SM300) was calculated. Plotted values correspond to the average of three biological replicates, with their standard error represented by bars (mean ± SEM). Statistical analysis consisted in independent ANOVA and Tukey’s tests; values with different superscript letters have a statistically significant difference (*p*-value < 0.05).

Finally, we performed a molecular validation of our results in a subset of differentially expressed genes. We selected six up-regulated genes in SM60 directly related to nitrogen metabolism (*DAL1*, *DAL5*, *DAL80*, *GAP1*, *PUT4*, and *UGA4*), and one down-regulated gene (*BAP3*) as a control. We exchanged each ORFs in the four parental strains by the luciferase reporter gene, allowing us to measure the transcriptional activity of the selected genes (Figure 3). Using this approach, we validated the SM60-overexpression for all genes in at least one genetic background (Figure 3). Interestingly, *DAL80* showed the higher expression levels in the NA background (Figure 3), which is consistent with our RNAseq results where *DAL80* was one of the most overexpressed gene in SM60, although in that case the imposed allele was from SA origin (Supplementary Table 5). Altogether, our results suggest a contribution of the NA alleles in the adaptation of SGRP-4X population to low nitrogen environments.

### Allele Frequency Changes Favour Adaptation of the Yeast Population to Nitrogen-Limited Fermentations

**FIGURE 4:**
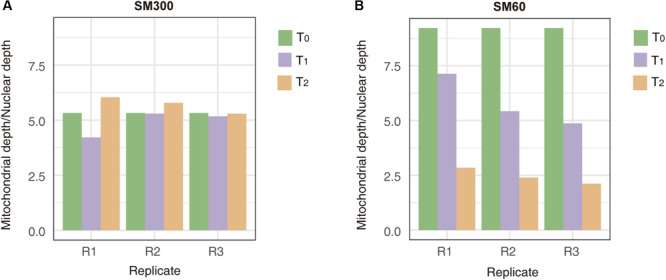
Mitochondrial genome copy number changes during the selection experiment. The mitochondrial depth/nuclear depth ratio are shown for experiments in **(A)** SM300 and **(B)** SM60. In each graph, the results for each experimental time point (*T*_0_, *T*_1_, or *T*_2_) at different replicas (*R*1, *R*2, or *R*3) are shown.

To obtain more insights related to the population adaptation to the nitrogen-limited fermentation condition and the importance of non-domesticated alleles in this process, we sequenced the DNA of all the samples obtained from selection experiments (*T*_0_, *T*_1_, and *T*_2_). The results from the average sequencing coverage showed that no aneuploidies appeared in the population across experiments (Supplementary Figure 9). In addition, the average allele frequency of the population was 0.25, suggesting that no clonal expansion took over the population during the selection process (Supplementary Figure 10). However, *de novo* mutations occurred in 13 different ORFs, where *de novo* mutation on *SSA2*, *RPL1A* and YNL018C appeared in different replicas and different time points of the selection experiment (Supplementary Table 6). Overall, *de novo* mutations were dominant over aneuploidies and appearance of clonal populations in the selection experiment, despite the low number of generations completed by the population. Interestingly, we also observed a loss of mitochondrial genetic material over the time course of the selection experiment in SM60, but not in SM300 (Figure 4). This loss in the mitochondrial genome is a general phenomenon, independent of the parental genetic background, since the allele frequencies of mitochondrial genomes were maintained through the selection experiment (Supplementary Figure 11).

**FIGURE 5:**
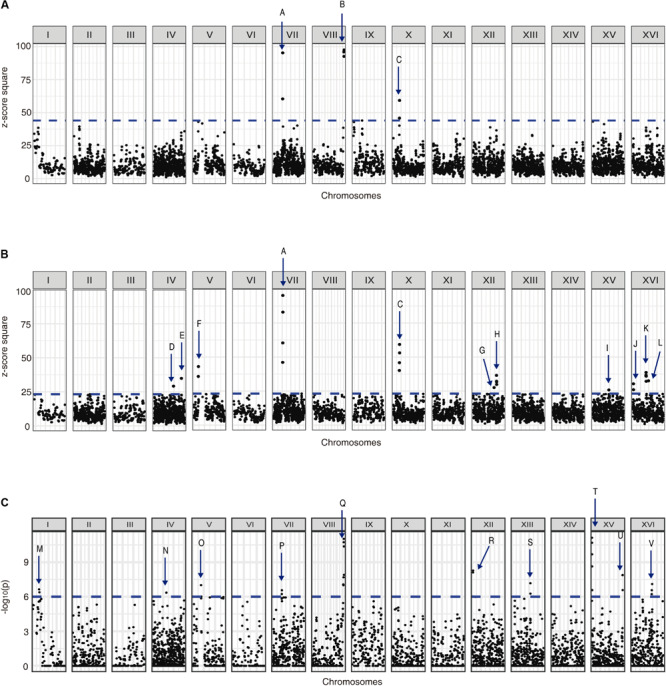
QTLs mapped for nitrogen-limited fermentations. Graphs shows **(A)** the *z*-score square for each genome position considering a 99% cut-off, **(B)** the *z*-score square for each genome position considering a 95% cut-off, and **(C)** the −log_10_(*p*) for each genome position considering a 99% cut-off. The mapped QTLs (A–V) are indicated with arrows.

We used the allele frequency changes across the selection experiments to map QTLs related to yeast adaptation under nitrogen-limited fermentation (Supplementary Figures 12, 13). Using a first approach based on *z*-scores, we identified 12 different QTLs in response to low-nitrogen conditions (named from A to L), from which three were strong QTLs (A–C, 99% cut-off) and nine were weak QTLs (D–L, 95% cut-off) (Figures 5A,B). Alternatively, using the −log_10_(*p*) approach we identified 10 QTLs (named from M to V) (Figure 5C), among which the strongest QTLs identified by our previous *z*-score approach were re-captured, where QTLs P and Q partially overlap with QTLs A and B, respectively (Figure 5). From the overall set of mapped QTLs, several genes were identified as candidate causative genes for the phenotype under study (Supplementary Tables 7–9).

An interesting candidate gene inside QTL G (Figure 5B) was *ECM38*, which encodes for a gamma-glutamyltranspeptidase, and it was also overexpressed in SM60 (Supplementary Table 2). Thereby, we performed a reciprocal hemizygosity analysis, generating the hemizygous strains for *ECM38* gene between the wine (WE) and wild (NA) strains. We observed that in low-nitrogen microculture fermentative conditions the hemizygous strain carrying the NA allele had a higher growth efficiency in comparison with the hemizygous strain carrying the WE allele (Table 3). This result reinforces the idea that alleles from the non-domesticated NA strain may favour yeast adaptation to nitrogen-limited fermentations.

**TABLE 3:** Growth parameters in the reciprocal hemizygous strains.

**Strain**	**Lag ± SD (h)**	**Rate ± SD (h^–1^)**	**Efficiency ± SD**
NA *ecm38*Δ × WE	1.99 ± 0.16	0.499 ± 0.012	0.873 ± 0.002*
NA × WE *ecm38*Δ	1.71 ± 0.11	0.493 ± 0.002	0.924 ± 0.009*

Statistical analysis of these parameters consisted in Welch two sample *t*-tests. Statistically significant differences between reciprocal hemizygous strains (*p*-value < 0.05) are shown with asterisks (*). SD, standard deviation.

## Discussion

In this work, we performed selection experiments in nitrogen-limited and nitrogen-sufficient wine fermentation conditions, using the SGRP-4X recombinant population, and then we sequenced the genome and transcriptome of the population at different time points across the selection process. Initially, we identified by RNAseq a set of 187 differentially expressed genes (“absolute differential expression log_2_ ratio” ≥2) in the low nitrogen condition (Supplementary Table 2). Among them, 38.1% correspond to nitrogen-related genes, which is superior to other RNAseq experiments performed in our laboratory. Recently, our group performed a RNAseq experiment using individual segregants derived from the SGRP-4X population in a non-limiting nitrogen condition (SM300) (Cubillos et al., 2017), where only 5.3% of the identified genes were nitrogen-related (when considering the same cut-off value). These results show the potential of our strategy that combines selection experiments (using two extreme nitrogen conditions, SM300, and SM60) with a highly recombined population for finding causative alleles for a phenotype of interest.

Our selection experiment set-up including two steps, batch and continuous fermentation, keeping constant the growth rate of the population and the selective pressure during the continuous culture fermentation step. Therefore, the doubling time of the population and the nutrients availability remains constant during this period (Figure 1). This set-up allowed us to obtain highly reproducible RNAseq and DNAseq results across biological replicas, confirming the robustness of chemostats in adaptative evolution experiments (Gresham and Hong, 2015). In a similar way, previously described selection experiments in bioreactors also have used nitrogen limitation as selective condition, identifying several genetic targets associated to nitrogen starvation such as CNV and *de novo* mutations (Gresham et al., 2010; Hong and Gresham, 2014; Lauer et al., 2018). However, these experiments have been restricted to clonal populations of the laboratory strain (S288c), reducing the genetic diversity of the yeast population under selection.

Interestingly, among the differentially expressed genes, there are groups of them with changes in gene expression consistent with those that have been previously reported, especially for genes under the control of the TORC1 signalling pathway (Vinod et al., 2008; Stephan et al., 2009; Nomura et al., 2010; Zhao et al., 2013). In particular, it has been reported that, in response to nitrogen starvation in wine alcoholic fermentation, nitrogen catabolic genes and stress responsive genes are activated, while genes related to amino acid biosynthesis, protein synthesis initiation and mRNA translation are repressed (Tesniere et al., 2015), which is corroborated by our results (Supplementary Tables 2–5). Moreover, recent evidence supports the existing link between nitrogen consumption, TORC1 activation and fermentative capacities in yeast (Kessi-Perez et al., 2019a; Molinet et al., 2019).

In general, the obtained results also indicate that, despite yeast cells are being in favourable conditions for the fermentation process (Table 2), in a nitrogen-limited condition they attempt to respire instead of fermenting, which is consistent with previous works (Tesniere et al., 2015). First, many genes involved in thiamine biosynthesis and uptake (*PHO3*, *THI4*, *THI5*, *THI11*, *THI12*, *THI13*, *THI20*, and *THI72*) are down-regulated in SM60, being thiamine pyrophosphate (TPP) a coenzyme of pyruvate decarboxylase that is required in the first step of alcoholic fermentation. This is consistent with the observation that during transfer from anaerobic to aerobic conditions, both mitochondrial and cytoplasmic thiamine contents fall and decarboxylase activity decreases (Suomalainen and Rihtniemi, 1962).

Moreover, a couple of genes that are known to be expressed in anaerobic and repressed in aerobic conditions are also down-regulated (*ANB1*, *DAN1*, and *TIR1*). On the other hand, genes involved in uptake and utilization of proline (*PUT1*, *PUT2*, and *PUT4*) are up-regulated in SM60, although proline is generally not consumed under fermentative conditions. Finally, one of the subunits of the mitochondrial pyruvate carrier (MPC) that is expressed during growth on fermentable carbon sources (*MPC2*) is down-regulated in SM60. Similarly, other subunit of the MPC that is expressed during growth on non-fermentable carbon sources (*FMP43*) is up-regulated in SM60, despite that the carbon sources used in the experiments (glucose and fructose) are both fermentable.

This apparent down-regulation of fermentation-related genes and up-regulation of respiration-related ones may be also related to the loss of mitochondrial genetic material observed over the selection experiment in SM60 (Figure 4). Moreover, various genes related to mitochondrial stability are down-regulated in SM60, such as: *THI4*, required for mitochondrial genome stability in response to DNA damage (Machado et al., 1997); *THI73*, whose transcription is altered in response to mitochondrial dysfunction (Epstein et al., 2001); *DLD3*, whose expression is stimulated by damage to mitochondria (Chelstowska et al., 1999); and *ICY2* (also known as *ATG14*), which is required for autophagy and mitophagy (Yao et al., 2015). However, other genes associated with respiration, such as the ones controlled by the Hap4p transcription factor, were not detected in our experiments (Zampar et al., 2013).

Importantly, when we focused our attention in a subset of differentially expressed genes, alleles coming from NA strain were predominantly overexpressed in nitrogen-limited conditions (Figure 2). This is consistent with the observation that the NA strain showed an apparently higher *μ*_max_ in SM60 batch cultures compared to the WE strain (Supplementary Figure 1 and Supplementary Table 1). One possible explanation to this phenomenon is that, while grape musts are usually supplemented with ammonium salts to overcome problems in nitrogen content (Garde-Cerdán and Ancín-Azpilicueta, 2008), non-domesticated strains are forced to adapt to environments where nitrogen can be a limiting nutrient (Ibstedt et al., 2015). However, more experimental evidence is necessary to check if NA strain is actually adapted to nitrogen limitations, or if this higher expression of nitrogen-related genes from NA origin may be indicative of other molecular mechanisms, such as general stress response, rather than low-nitrogen adaptation *per se*.

Moreover, we molecularly validated *DAL80*, one of most overexpressed gene in SM60 (Supplementary Tables 1, 2, and 5), which showed the highest transcriptional activity (measured as luciferase reporter gene expression) in the NA background (Figure 3). This gene encodes a negative regulator of genes in multiple nitrogen degradation pathways and its expression is regulated by nitrogen levels (Marzluf, 1997; Magasanik and Kaiser, 2002), being very important for adaptation to low-nitrogen conditions. In particular, DAL80p is a central component of the nitrogen catabolite repression (NCR), a nitrogen regulation of transcription that, although is directly regulated by the TORC1 pathway, is functionally distinct from it (Ljungdahl and Daignan-Fornier, 2012).

Besides *DAL80*, no other central gene of the NCR (e.g., *GAT1*, *GLN3*, *GZF3*, and *URE2*) pathway was identified among the differentially expressed genes, neither are any of the central genes of other important mechanisms of nitrogen metabolism regulation such as the SPS system, the general amino acid control (GAAC) or the retrograde pathway (RTG) (Supplementary Table 2). However, there are various genes that are experimentally verified (*DAL1*, *DAL2*, *DAL7*, *DCG1*, *DUR1*, *2*, *PUT1*, *PUT2*, *AGP1*, *DAL4*, *DAL5*, *DUR3*, *GAP1*, *MEP2*, *MEP3*, *PUT4*, *UGA4*, *DAL80*, *1*, *PRB1*, *ECM38*, and YGR125W) or predicted (*NIT1*, *OPT2*, *VBA1*, *GUD1*, *LEE1*, *RPS0B*, *UGX2*, *DSD1*, and YLR257W) to be NCR-controlled (Godard et al., 2007), encompassing categories of amino acid-nitrogen metabolism, plasma membrane nitrogen uptake, transcription factors, vacuole function, nucleotide salvage pathways, and others (Ljungdahl and Daignan-Fornier, 2012). This is in agreement with previous observations where NCR normally ensures that yeast cells use preferred nitrogen sources selectively when they are available, while general de-repression of NCR-regulated genes enables them to use non-preferred nitrogen sources in the absence of preferred ones (Ljungdahl and Daignan-Fornier, 2012).

The importance of wild alleles from the non-domesticated strains in adaptation to nitrogen-limited fermentation conditions is also suggested by the results obtained from the QTL mapping. Using the alleles frequencies obtained from the DNAseq analysis of the population under selection, we mapped several QTLs for fermentation under a nitrogen-restricted condition using two different approaches, *z*-scores and −log_10_(*p*) values (Figure 5). Interestingly, while the strongest QTLs identified by the *z*-score approach were also captured by the second approach, there were also other non-overlapping QTLs, which is indicative that weak QTLs may be affected by the mapping approach used. Moreover, we validated by reciprocal hemizygosity analysis that the NA allele of *ECM38* gene, a gene that also is up-regulated in SM60 (Supplementary Tables 1, 2), causes a higher efficiency of proliferation compared to the WE allele in SM60 (Table 3), giving indirect support to its role in low-nitrogen fermentative adaptation. *ECM38* encodes a gamma-glutamyltranspeptidase, and its expression is known to be induced mainly by nitrogen starvation (Springael and Penninckx, 2003), which is consistent with the results obtained in our RNAseq experiments.

Interestingly, *ECM38* was identified from both RNAseq and DNAseq analyses, suggesting that expression levels and allele frequency changes of this gene could be important for adaptation of the yeast population to nitrogen-limited fermentation. However, due to its role in oxidative stress and xenobiotic detoxification (Ubiyvovk et al., 2006), this adaptation to nitrogen limitation may be consequence of the stress response mechanisms in the NA strain. Nonetheless, and regardless of the specific molecular mechanisms involved, altogether our results reinforce the idea that alleles coming from a non-domesticated strain (NA) may favour adaptation to low-nitrogen conditions, in this case of a heterogeneous yeast population, as we discussed recently (Kessi-Perez et al., 2020). However, more experimental evidence is necessary to confirm if NA strain is actually adapted to nitrogen limitations and if wild alleles may favour adaptation to nitrogen-limited fermentations. In conclusion, the selection experiments under nitrogen-restricted fermentation conditions, using a heterogeneous yeast population founded by four highly divergent strains, allowed us to identify several genetic signatures that may favour population adaptation. Importantly, alleles coming from a wild, non-domesticated isolate (NA strain) were overexpressed, suggesting an important role of this genetic background in the adaptation to nitrogen-limited fermentative conditions. These results highlight the potential biotechnological uses of non-domesticated yeast strains in the genetic improvement of wine yeast strains, enhancing their adaptation to grape musts with nitrogen deficiencies.

## Data Availability Statement

The DNA and RNA sequencing information used in this work is available in the SRA repository (https://www.ncbi.nlm.nih.gov/sra) [BioProject ID: PRJNA606912]. All other relevant data are within the manuscript and it’s Supplementary Material.

## Author Contributions

EK-P, AD-B, FS, and CM: conceptualization and validation. EK-P, JL, and FS: data curation, formal analysis, and writing – original draft preparation. CM: funding acquisition and project administration. EK-P, BP, JL, JM, CBae, DF, CBas, MG, and FS: investigation. EK-P, JL, AD-B, GL, FS, and CM: methodology. AD-B, GL, FS, and CM: resources. JL: software. FS and CM: supervision. EK-P and FS: visualization. EK-P, BP, JM, JL, AD-B, FS, and CM: writing – review and editing.

## Conflict of Interest

The authors declare that the research was conducted in the absence of any commercial or financial relationships that could be construed as a potential conflict of interest.

## Footnotes

1 www.yeastgenome.org

2 http://bioinformatics.sdstate.edu/go/
