# Supplementary figures and images for: Differential Gene Expression and Allele Frequency Changes Favour Adaptation of a Heterogeneous Yeast Population to Nitrogen-Limited Fermentations
Source: Front Microbiol. 2020 Jun 15;11:1204. doi: 10.3389/fmicb.2020.01204 (PMC7307137; doi:10.3389/fmicb.2020.01204)

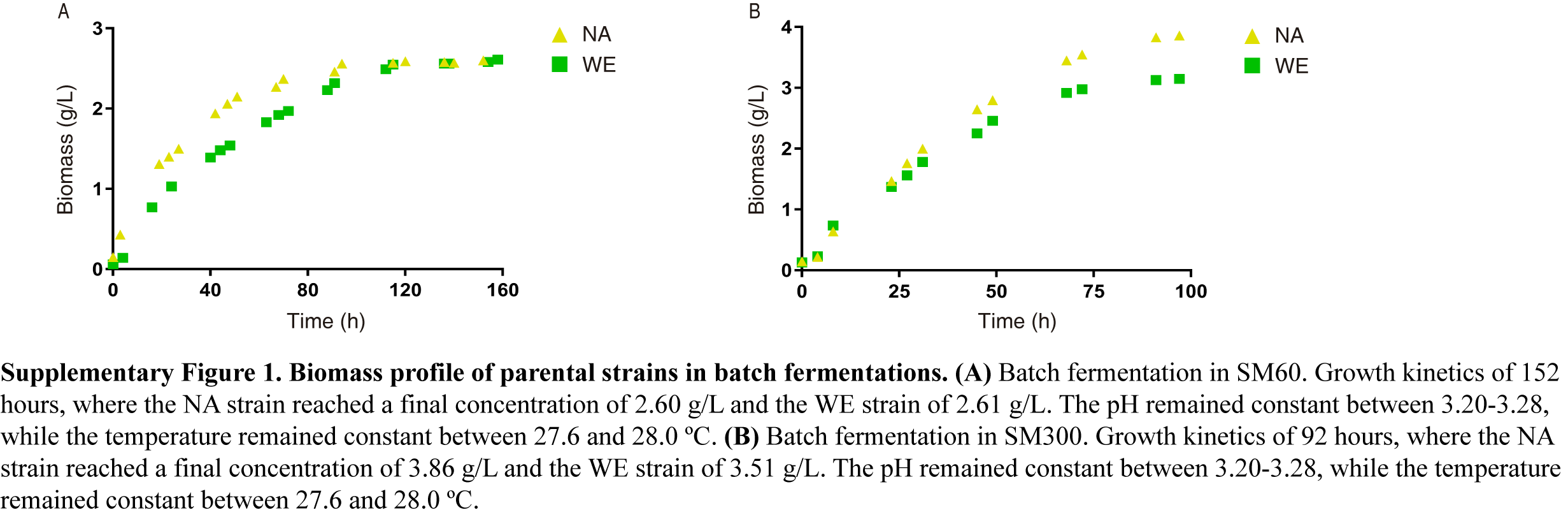

Supplement: Supplementary file 10 [file Image_1.TIF]

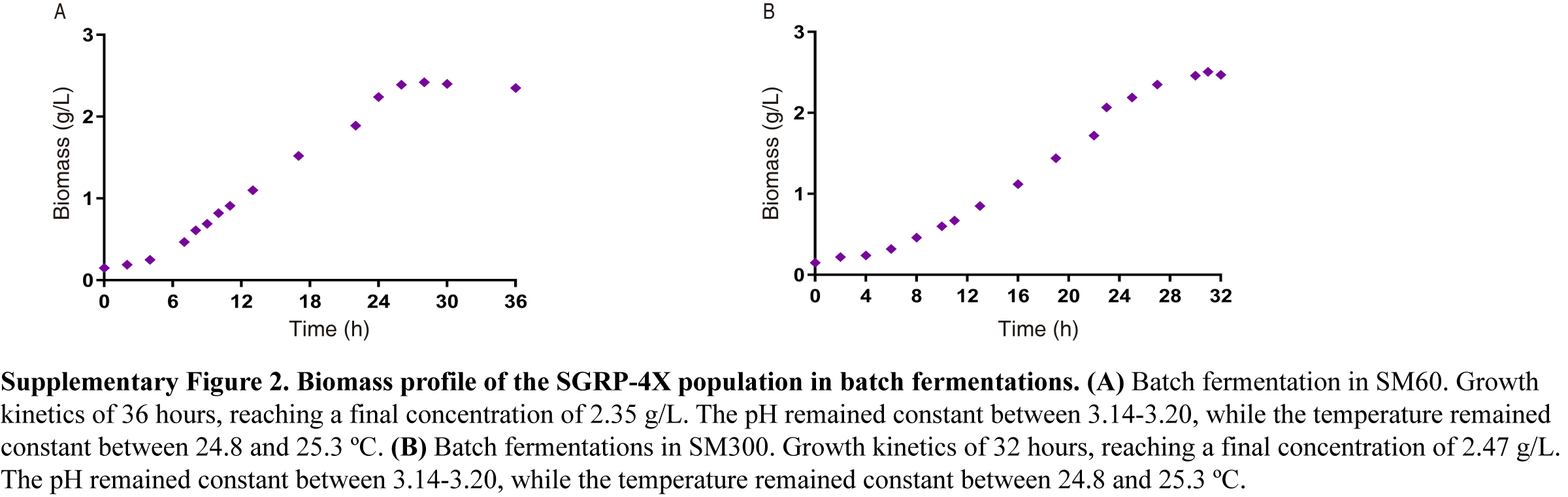

Supplement: Supplementary file 11 [file Image_2.TIF]

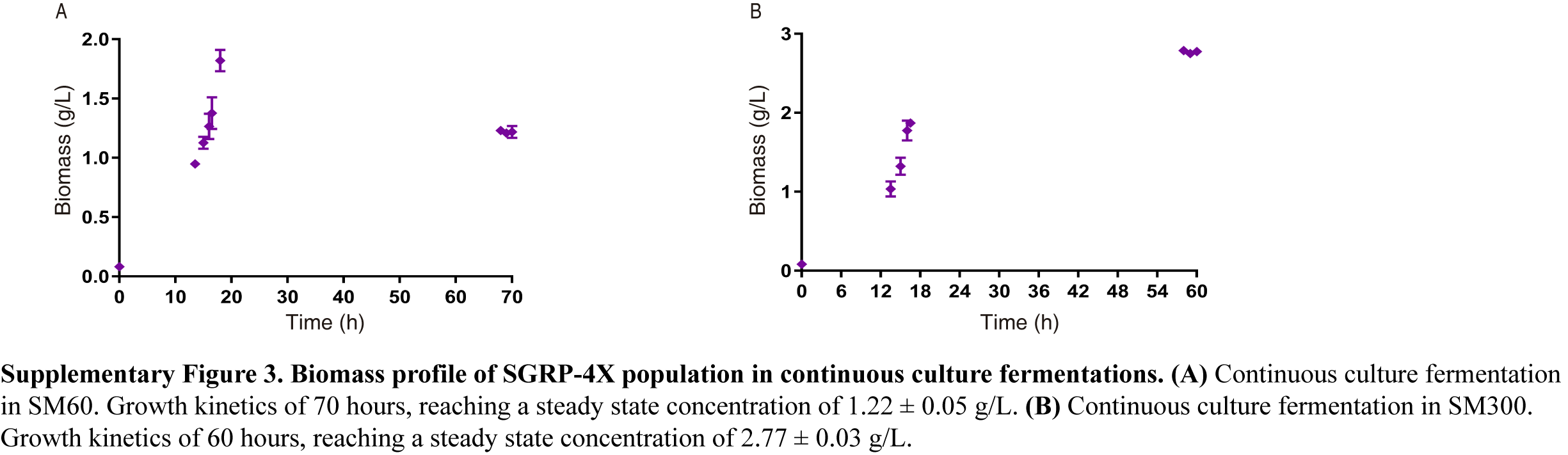

Supplement: Supplementary file 12 [file Image_3.TIF]

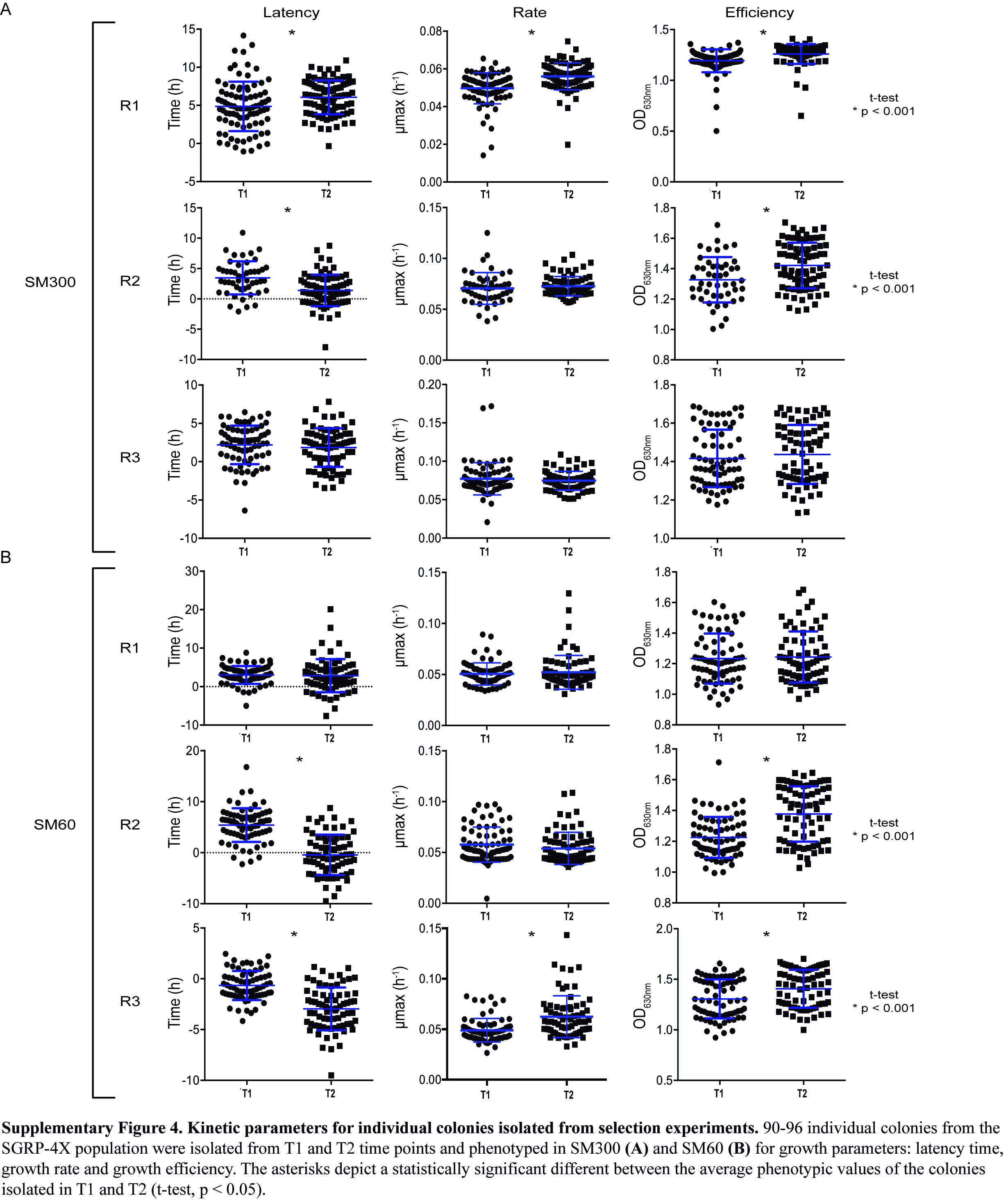

Supplement: Supplementary file 13 [file Image_4.TIF]

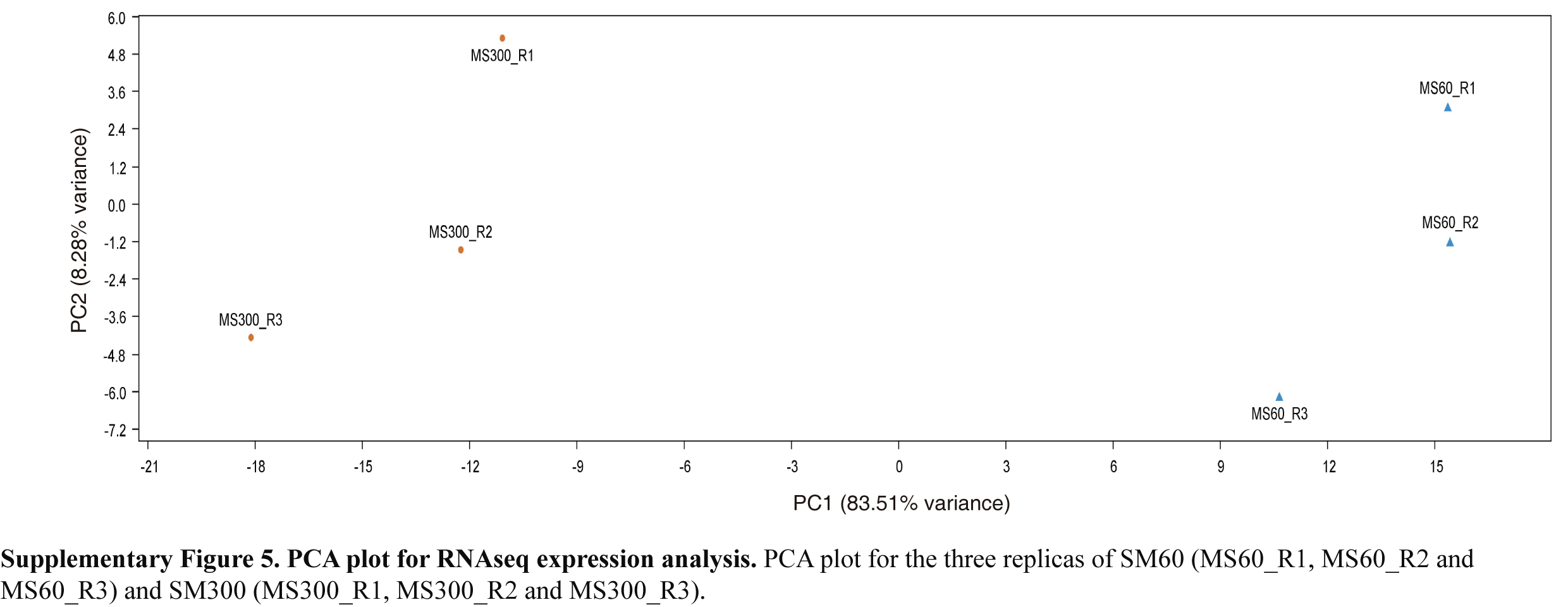

Supplement: Supplementary file 14 [file Image_5.TIF]

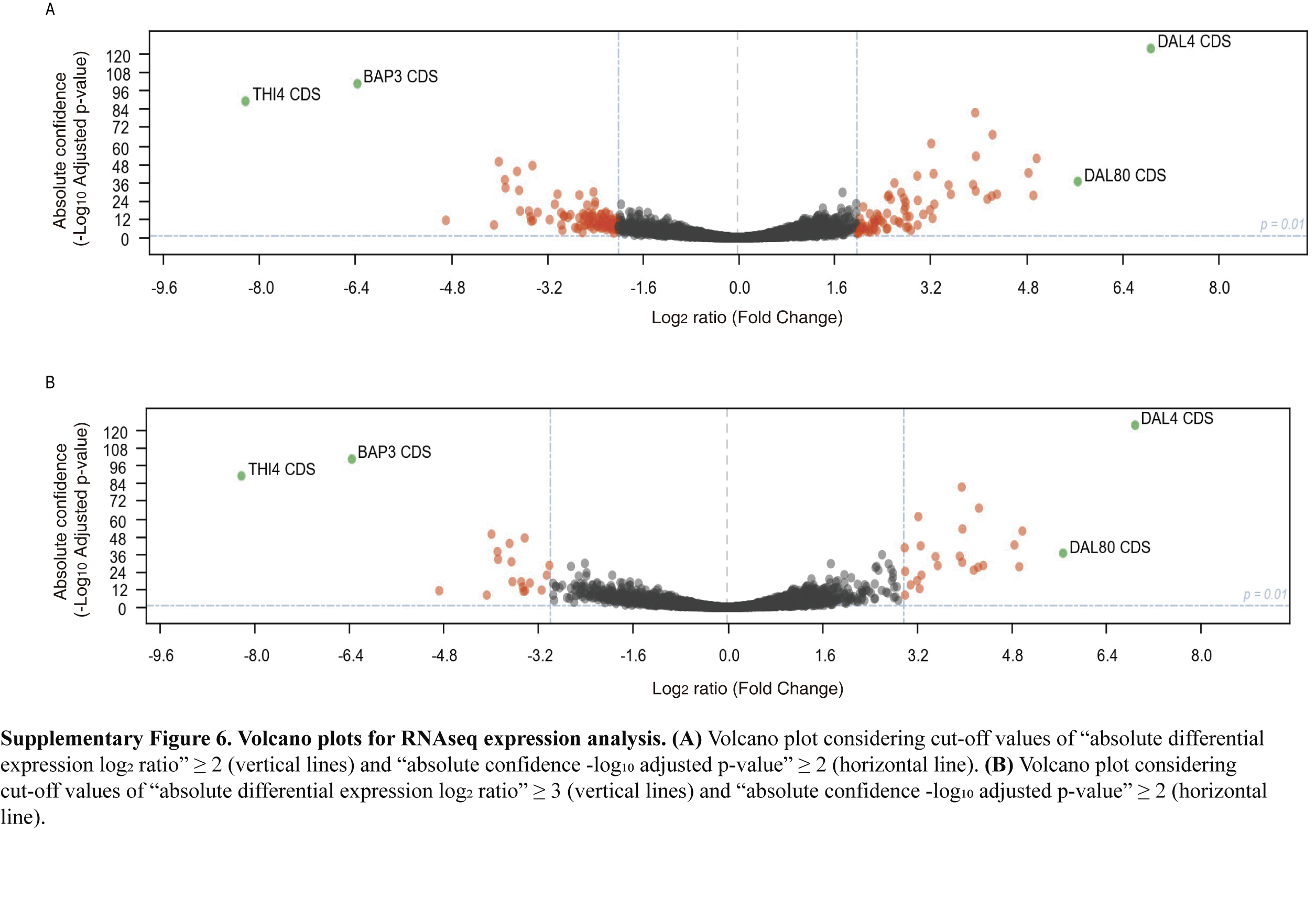

Supplement: Supplementary file 15 [file Image_6.TIF]

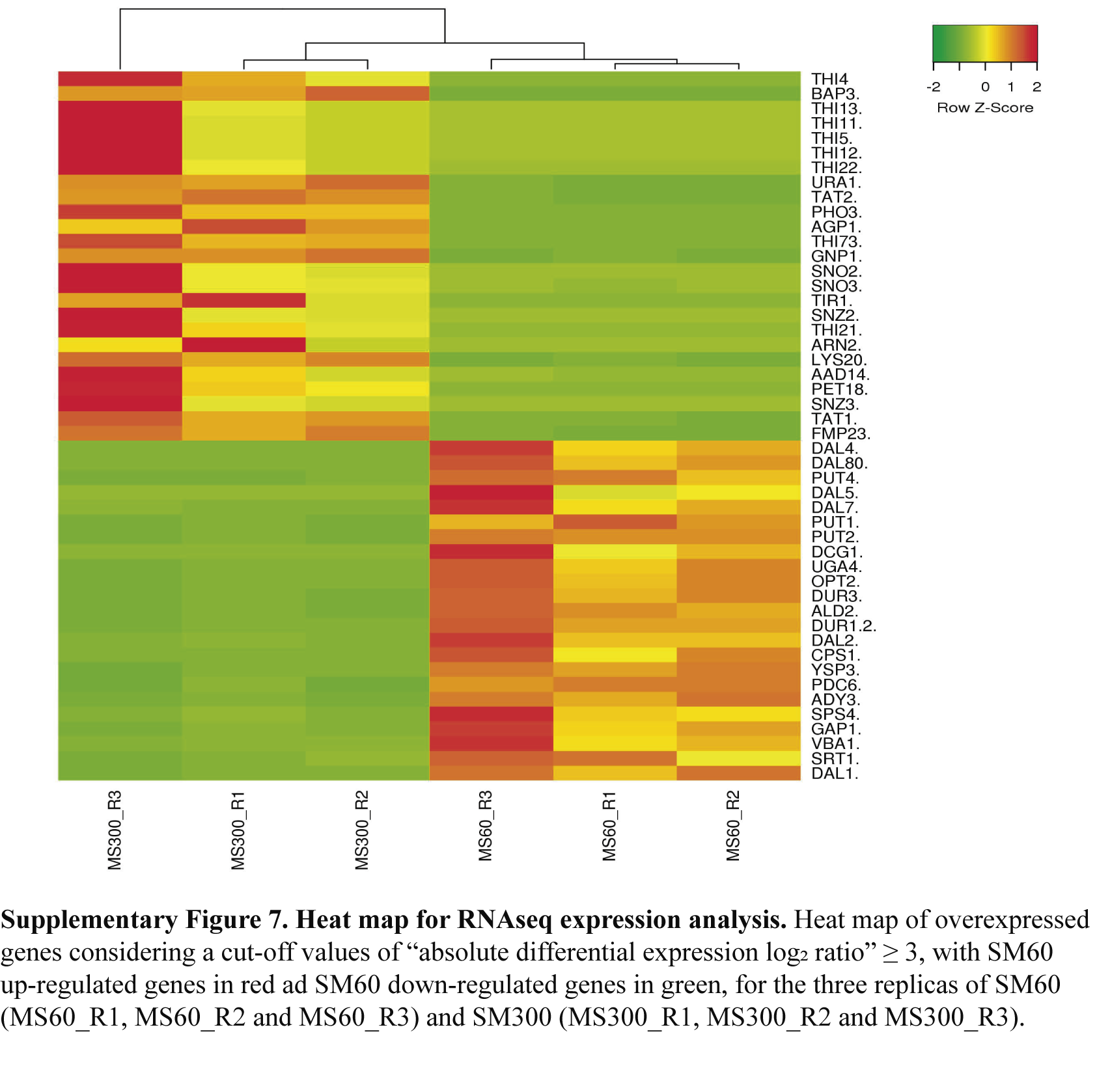

Supplement: Supplementary file 16 [file Image_7.TIF]

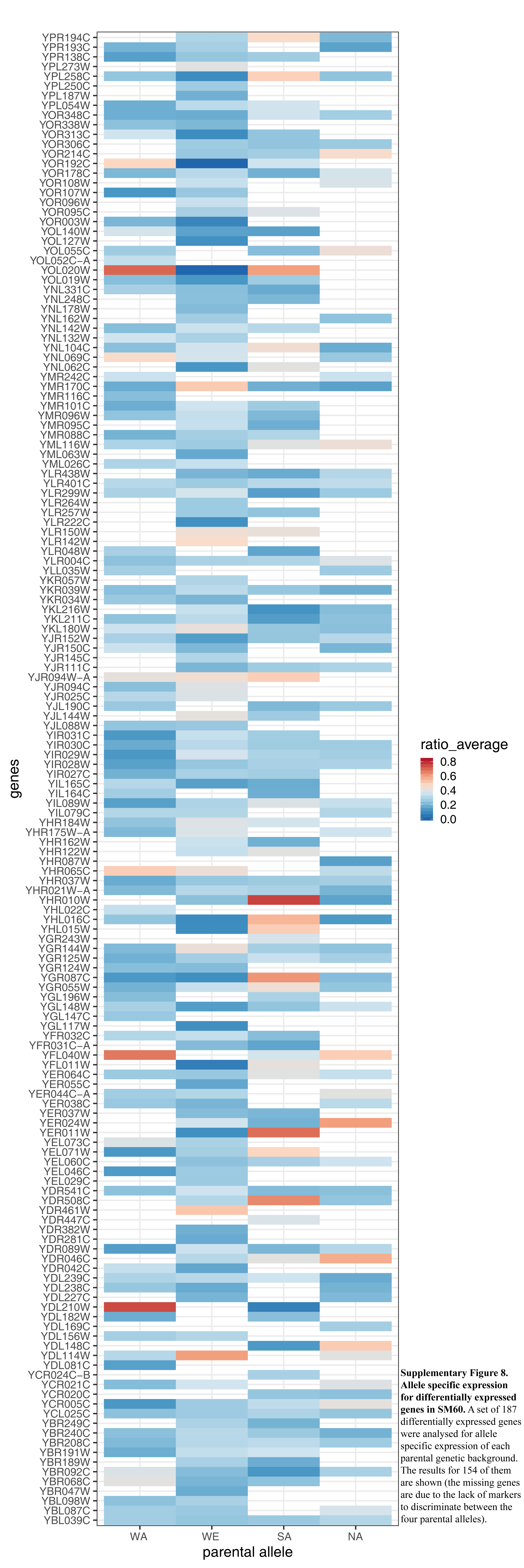

Supplement: Supplementary file 17 [file Image_8.TIF]

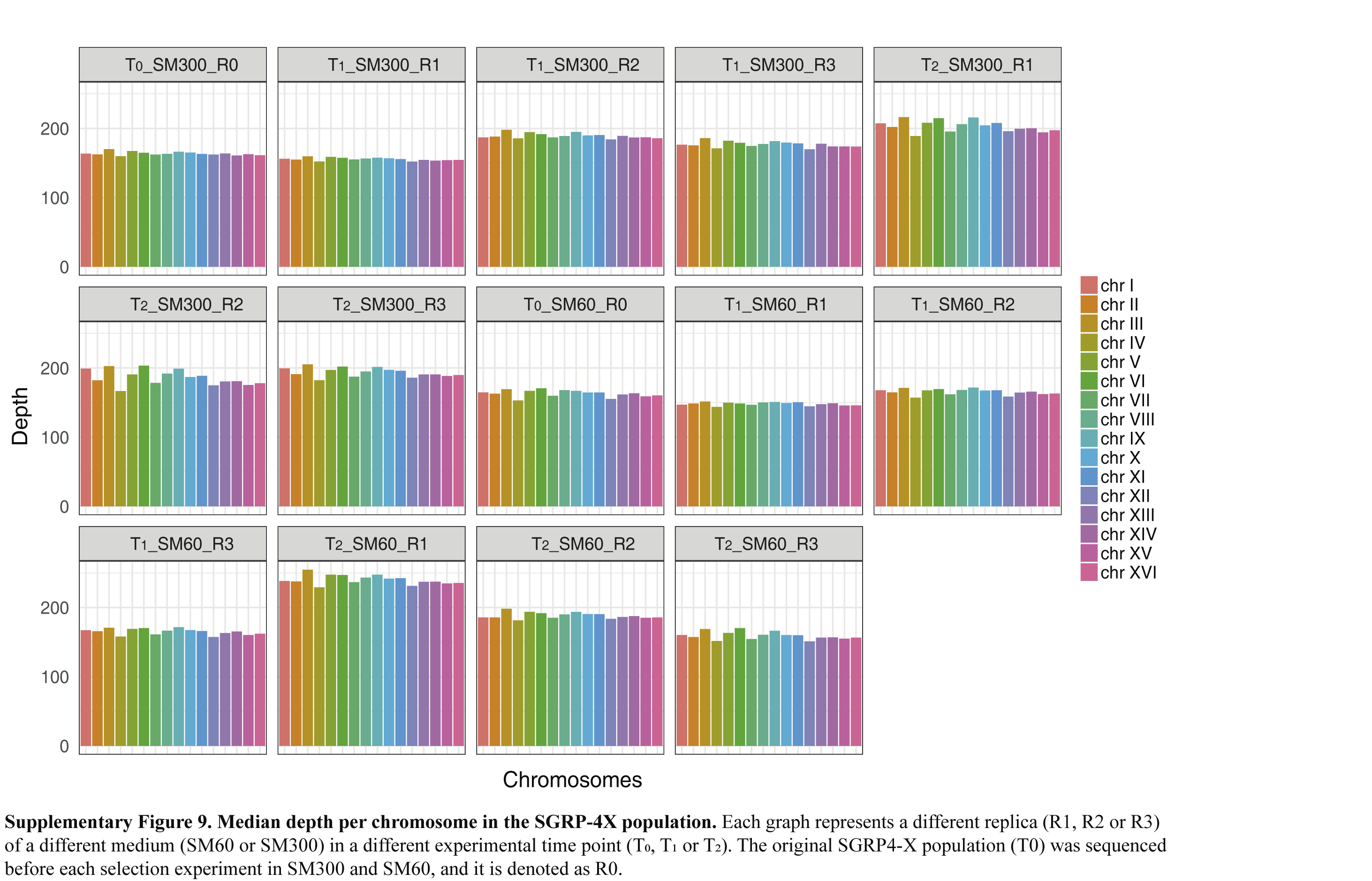

Supplement: Supplementary file 18 [file Image_9.TIF]

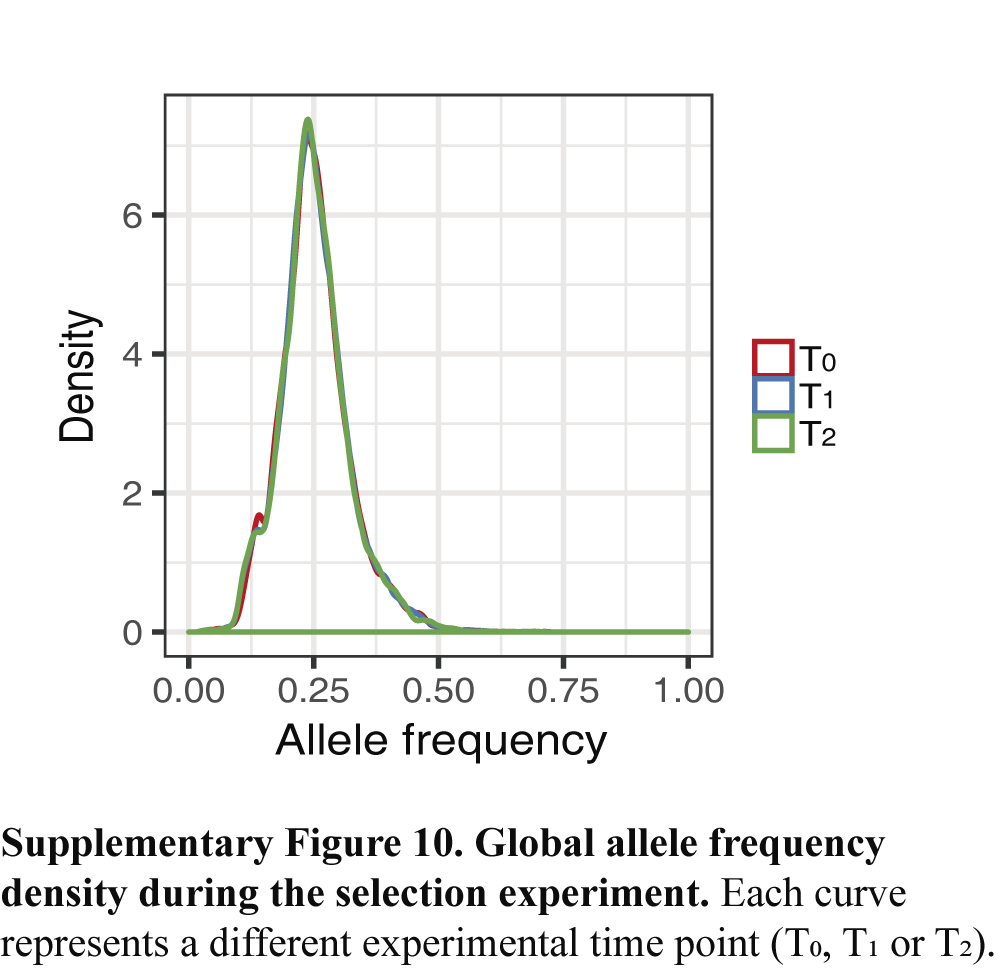

Supplement: Supplementary file 19 [file Image_10.TIF]

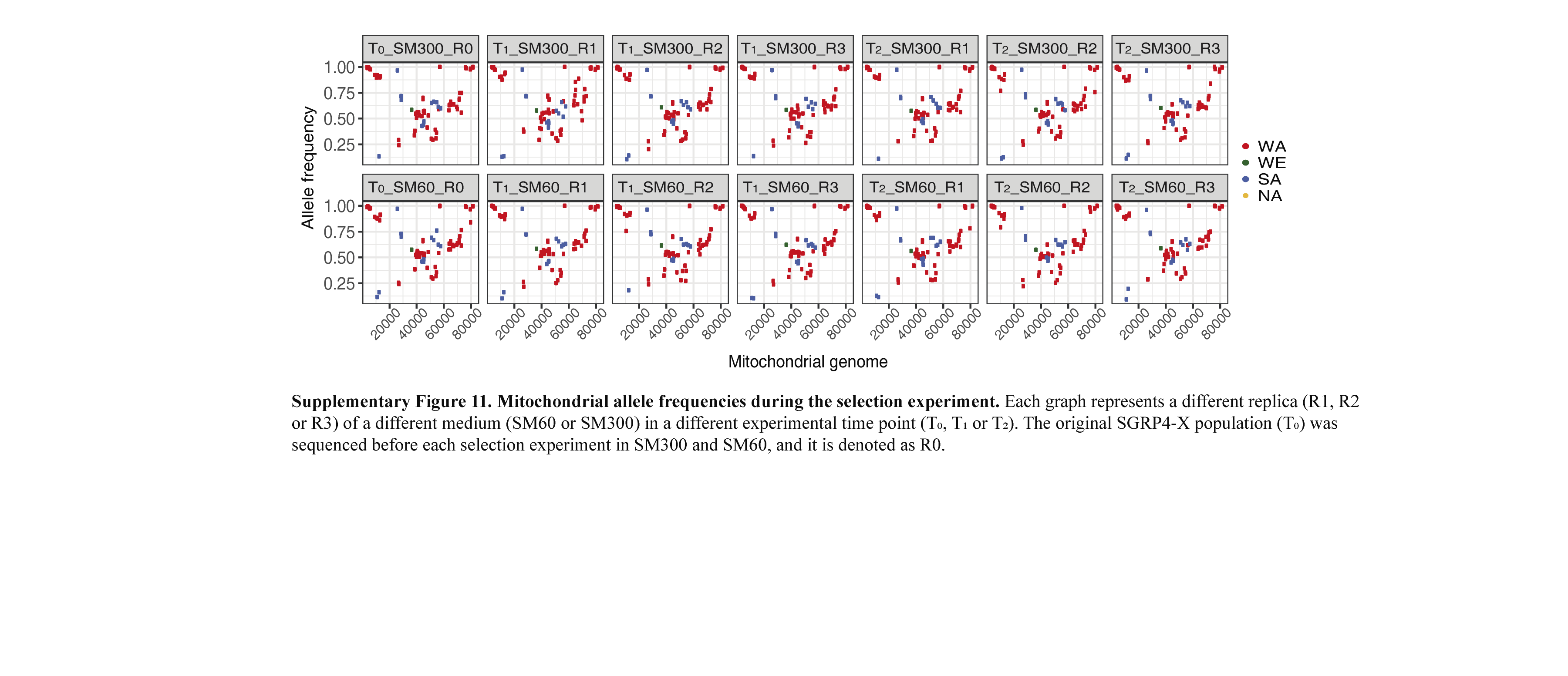

Supplement: Supplementary file 20 [file Image_11.TIF]

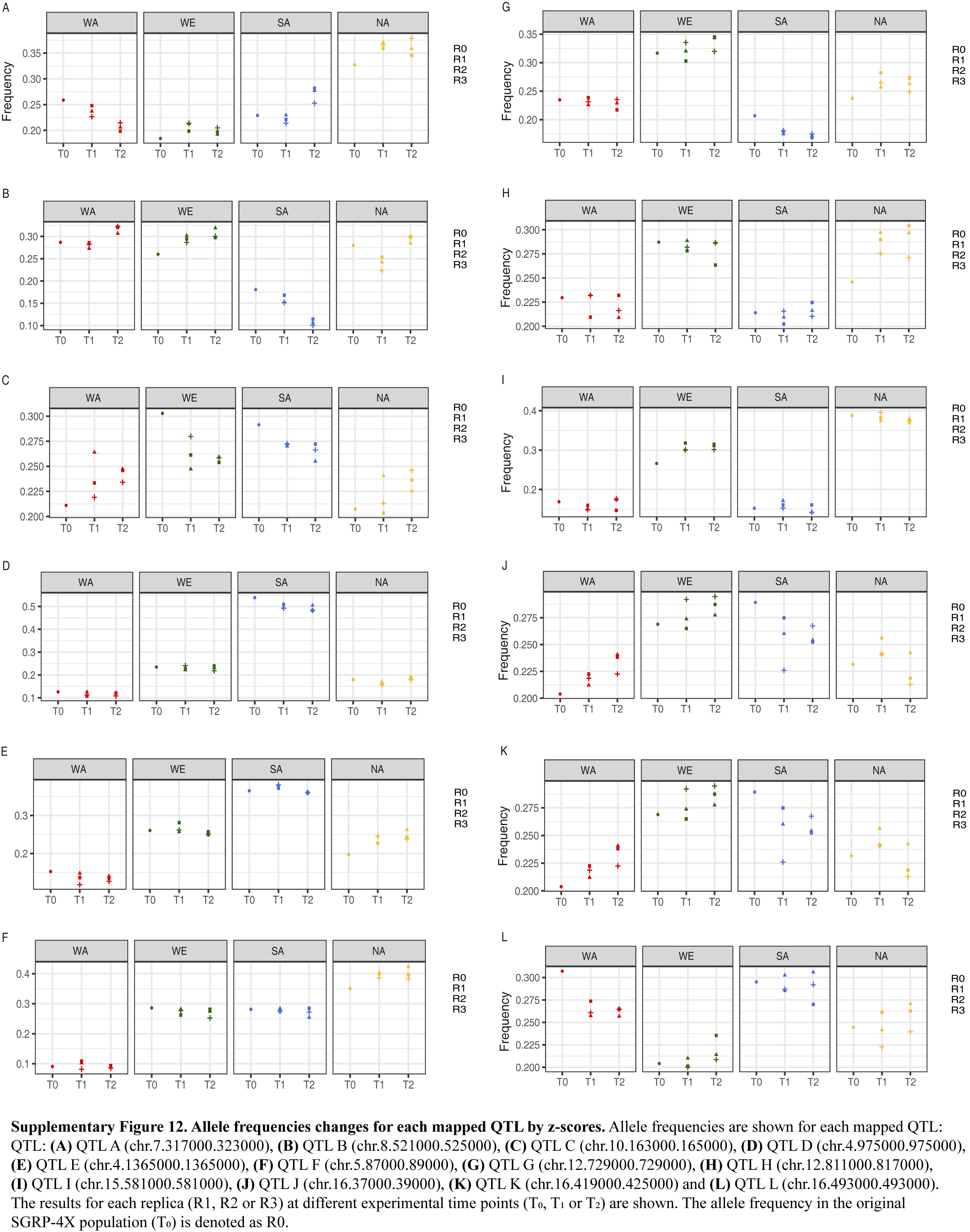

Supplement: Supplementary file 21 [file Image_12.TIF]

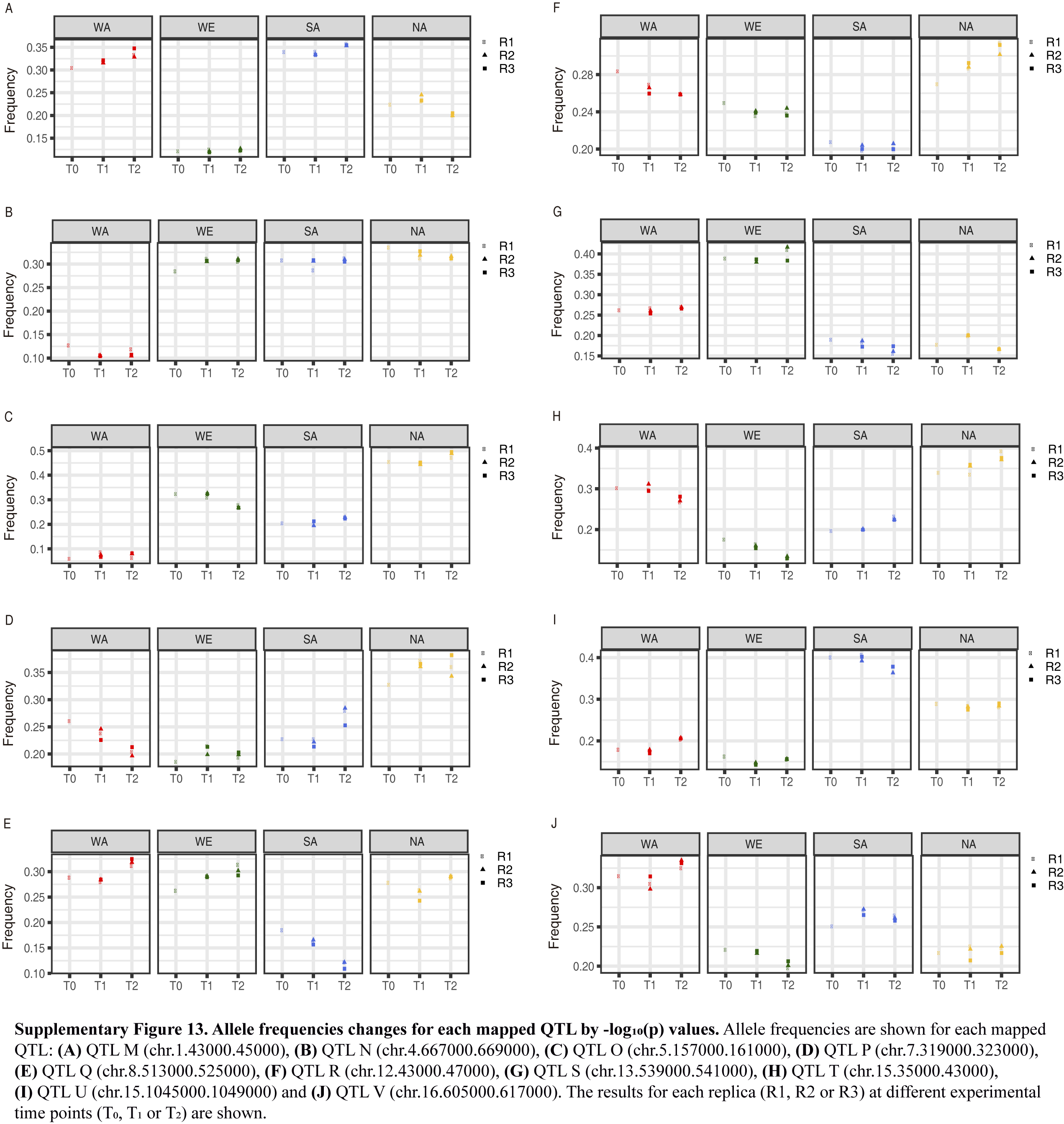

Supplement: Supplementary file 22 [file Image_13.TIF]
